# Forward Genetic Screens as Tools to Investigate Role and Mechanisms of EMT in Cancer

**DOI:** 10.3390/cancers14235928

**Published:** 2022-11-30

**Authors:** Ákos Gasparics, Attila Sebe

**Affiliations:** 1 Department of Obstetrics and Gynecology, Semmelweis University, 1088 Budapest, Hungary; 2 Division of Medical Biotechnology, Paul Ehrlich Institute, Paul-Ehrlich-Str. 51-59, 63225 Langen, Germany

**Keywords:** epithelial–mesenchymal transition, forward genetic screen, insertional mutagenesis screen, cancer functional genomics

## Abstract

**Simple Summary:**

Forward genetic screens link genome modifications to phenotypes, and have been successfully employed to identify oncogenes, tumor suppressor genes and genes involved in metastasis or therapy resistance. In particular, transposon-based insertional mutagenesis screens and CRISPR-based screens are versatile and easy-to-use tools employed in recent years to discover and identify novel cancer-related mechanisms. In this review, we present the contribution of forward genetic screens to our understanding of how EMT is regulated and how it is involved in various aspects of cancer.

**Abstract:**

Epithelial–mesenchymal transition (EMT) is a process of cellular plasticity regulated by complex signaling networks. Under physiological conditions, it plays an important role in wound healing and organ repair. Its importance for human disease is given by its central role in chronic fibroproliferative diseases and cancer, which represent leading causes of death worldwide. In tumors, EMT is involved in primary tumor growth, metastasis and therapy resistance. It is therefore a major requisite to investigate and understand the role of EMT and the mechanisms leading to EMT in order to tackle these diseases therapeutically. Forward genetic screens link genome modifications to phenotypes, and have been successfully employed to identify oncogenes, tumor suppressor genes and genes involved in metastasis or therapy resistance. In particular, transposon-based insertional mutagenesis screens and CRISPR-based screens are versatile and easy-to-use tools applied in recent years to discover and identify novel cancer-related mechanisms. Here, we review the contribution of forward genetic screens to our understanding of how EMT is regulated and how it is involved in various aspects of cancer. Based on the current literature, we propose these methods as additional tools to investigate EMT.

## 1. The Role of EMT in Cancer

Epithelial–mesenchymal transition (EMT) is defined as a process in which epithelial cells lose their characteristics and acquire mesenchymal properties. This translates into a loss and a gain of function for the affected cells. Cuboidal morphology, cell–cell junctions, apicobasal polarity and epithelial marker expression are lost during the process. In parallel, cells acquire a spindle shape and mesenchymal marker expression, and show increased motility and an activated myogenic program. During embryonic development, both EMT and mesenchymal–epithelial transition (MET) are required for cellular organization and organogenesis [1].

After birth, EMT is associated with wound healing and organ repair under physiological conditions. However, under pathological conditions, EMT plays an important role in supporting progressive disease. For example, a continuous activation of the program in a chronic inflammatory background may lead to organ fibrosis [2]. In malignant tumors, EMT plays important roles in all stages of cancer progression, from initiation, primary tumor growth, invasion, dissemination and metastasis to colonization; it is also involved in therapy resistance [3,4]. Various transcriptional factors and complex signaling networks regulate EMT. During the mainly TGF-β-dependent process, cancer cells start to express various mesenchymal markers (e.g., N-cadherin, vimentin), while the epithelial expression pattern is downregulated (e.g., E-cadherin) [5,6]. Following the initial concepts of terminal epithelial and mesenchymal phenotypes, EMT currently represents dynamic states of cellular fate, and the concept of partial EMT or epithelial–mesenchymal plasticity was introduced to describe cells simultaneously presenting both epithelial and mesenchymal traits [4,7].

The role of EMT throughout the metastatic cascade is extensively studied. According to the reversible EMT model, tumor cells activate the EMT program to achieve local invasion and dissemination to distant organs. These mesenchymal cells may revert via MET to an epithelial identity in the distant organ sites. Cells may regain their proliferative ability there, and their potential to form epithelial growths. This rather “classical”, complete EMT may coexist with cells concomitantly expressing epithelial and mesenchymal markers characteristic of an incomplete, partial EMT [8]. Single cell migration occurs following the acquisition of a complete mesenchymal phenotype, while the hybrid epithelial/mesenchymal phenotype ensures collective cell migration, which may also correlate with enhanced stemness and high tumor initiation capacity [9]. Circulating breast cancer cells were shown to exhibit dynamic changes in epithelial and mesenchymal traits correlating to clinical observations on disease progression and treatment response [10]. Moreover, the generation of bi-specific antibodies, concomitantly recognizing both epithelial (E-cadherin) and mesenchymal (OB-cadherin) targets, allow the identification of circulating tumor cells [11]. In the primary tumors, such cells with a mixed epithelial/mesenchymal phenotype may protect their epithelial neighbors from immune attack [12].

EMT is also involved in mechanisms related to therapy resistance [13]. For example, IL-6 and treatment with cisplatin increased TGF-β expression, and the subsequent EMT conferred resistance to cisplatin in NSCLC cells [14]. Oncostatin M may induce the expression of Zeb1 and Snail (SNAI1), thereby regulating an EMT program conferring resistance to gemcitabine in pancreatic cancer [15]. The co-expression of the N-cadherin or vimentin with PD-L1 was detected in circulating tumor cells (CTC) of recurrent patients treated with nivolumab, a PD-L1 inhibitor. PD-L1-positive CTCs isolated from NSCLC patients are characterized by a partial EMT phenotype. The co-expression of PD-L1 and EMT markers might represent a possible molecular background for immune escape [16], and hybrid epithelial/mesenchymal cells may express levels of PD-L1 high enough to become immune-evasive [17]. Importantly, EMT plays a defining role in resistance to radiotherapy as well [18].

## 2. Forward Genetic Screens in Cancer Research

With the wide availability of technology, large-scale, high-throughput tumor sequencing projects were conducted in recent years to comprehensively analyze genetic alterations in cancer genomes. While a multitude of recurrent mutations and genetic alterations were identified, deciphering the functional role of these changes proved to be difficult, and discerning driver from passenger mutations is still a challenge [19]. Several high-throughput, genome-wide screening methods were developed for cancer functional genomics to identify tumor suppressors and oncogenes, such as insertional mutagenesis, overexpression of cDNA/ORF libraries or RNAi- and CRISPR-based screens. These forward genetic screening methods provide a significant advantage by linking genome modifications to phenotypes, enabling a direct investigation of gene functions, and have been employed to identify oncogenes, tumor suppressor genes, genes involved in metastasis or therapy resistance and tumor cells of origin [20,21,22,23].

While most of these methods rely on the design of predefined libraries, insertional mutagenesis has the advantage of a random, genome-wide distribution of the insertional mutagens. Retroviruses, lentiviruses and transposons were used to induce cancer by modifying the genes hosting their integrations by different mechanisms: enhancing transcription or translation levels of genes, inactivating gene expression or by generating chimeric or truncated transcripts. Retrovirus-based screening experiments took advantage of the fact that retroviruses cause lymphomas and mammary tumors: the random proviral insertions can cause activation or inactivation of genes, resulting in neoplasias. Similarly, lentiviruses can induce insertional mutagenesis by both inducing gain-of-function and loss-of-function mutations with a certain bias towards integration within actively transcribed genes, and can interact with the splicing machinery of the host gene, resulting in the generation of truncated transcripts [24].

The most widely used tools for insertional mutagenesis-based forward genetic screens are transposons. The Sleeping Beauty [25,26] and piggyBac [27] DNA transposons were designed to integrate cassettes containing a promoter and a splice donor, as well as two splice acceptor sites to generate gain-of-function and loss-of-function mutations. Transposon mutagenesis has been used to interrogate tumorigenesis and cancer genes in a multitude of cancer types in mice [20,24,28].

Besides transposon-based forward genetic screens, in recent years, the CRISPR/Cas9 system also evolved into a frequently used, powerful cancer screening tool [23,29]. For CRISPR screens, the designed single-guide RNAs (sgRNA) are cloned into lentivirus libraries and transduced into Cas-expressing cells to obtain single-copy sgRNA integrations [30]. In addition to knockout of coding genes by the generation of targeted double-strand DNA breaks, the CRISPR technology was developed as CRISPRi/CRISPRa to repress or activate the transcription of protein-coding genes by targeting repressor or activator mediators to the transcriptional start sites [31].

These functional genomics methods were applied in several studies interrogating the roles and mechanisms of EMT and epithelial mesenchymal plasticity in cancer. For example, a retroviral cDNA library was used to identify new metastasis-promoting genes in an in vivo breast cancer model: the thiol isomerase ERp5 was identified as an activator in the early steps of metastasis by activating the key EMT regulators PI3K, RhoA and β-catenin [32]. A gain-of-function screen using a lentiviral expression library of 17,000 human open reading frames (ORFs) identified genes that induce EMT in breast cancer [33]. An RNAi screen led to the identification of KLF17 as a negative regulator of EMT and breast cancer metastasis [34]. In an in vivo screen with breast cancer cells mutagenized with a replication-incompetent gammaretroviral vector, SHARPIN and RIN1 were described as novel breast cancer metastasis genes [35]. SHARPIN was identified as a novel adaptor protein to stabilize β-catenin and activate downstream signaling [36], whereas RIN1 was later shown to mediate EMT [37].

While these shortly presented screening methods were used successfully to identify EMT-related mechanisms, as mentioned above, transposon-based insertional mutagenesis screens and CRISPR-based screens are the most frequently used tools to identify novel cancer genes and mechanisms. Here, we review the contributions of these two easy-to-use and versatile screening tools to our understanding of the role of EMT in cancer and the regulatory mechanisms governing it (Table 1 and Table 2).

## 3. Transposon-Based Forward Genetic Screens and EMT

Breast cancer is the most commonly diagnosed cancer among women worldwide and is the leading cause of cancer-related deaths among women [48]. BRCA1, the first breast cancer susceptibility gene identified, is responsible for 5–10% of total breast cancers [49]. The Sleeping Beauty (SB) transposon system was used in BRCA1-deficient mice to identify cancer driver genes accelerating triple-negative breast cancer (TNBC) [38]. The SB tumorigenesis system markedly accelerated tumorigenesis by increasing tumor incidence and tumor burden. The screen identified 169 candidate genes correlating with tumorigenesis, of which Notch1 emerged as a top putative oncogene that overcomes apoptosis caused by Brca1 deficiency and promotes TNBC formation. When exploring the mechanisms related to Notch1-driven TNBC tumor formation, the authors found that genes upregulated in the EMT process were highly expressed, while genes downregulated during EMT showed low levels of expression in Notch1-driven tumors. Findings also indicated that NOTCH1 promotes EMT, leading to TNBC formation in BRCA1-defective conditions, correlating with human BRCA1-mutant breast cancer samples showing high levels of mesenchymal markers.

Human germline PTEN mutations also represent a lifetime risk for a variety of cancers, not only breast cancers [50]. In a screen to identify genes that co-operate with mutant Pten in the induction of TNBC, the SB mutagenesis tool was deployed in breast epithelial cells of mice that were heterozygous for a Pten-null allele [39]. Here again, SB mutagenesis accelerated mammary tumor formation in Pten^fl/+^ mice. The analysis of the transposon integration sites identified several candidate genes, of which Man1a1, Pkp4, Rab10, Rasa1, Trps1, Vps26a, Xpnpep3 and Znf326 accelerated tumor growth, whereas the silencing of R3hcc1l reduced tumor growth. Downregulation of Trps1 resulted in the largest acceleration of tumor growth in the functional validation studies, and TRPS1 functions as a tumor suppressor gene in TNBC. When TRPS1-overexpressing cells were injected into the mammary fat pad of athymic nude mice, a significant decrease in lung metastatic progression was observed, indicating that TRPS1 is a tumor suppressor gene that inhibits lung metastasis. Moreover, in correlation to the findings in the metastasis model, TRPS1 knockdown was shown to regulate the expression of multiple genes in the EMT pathway, leading to significant upregulation of BMP2, MMP2, MMP9, SERPINE1, SNAI2, TFPI2, TGFB2 and ZEB1 and a significant downregulation of COL5A, FN1, KRT14, SNAI1, SNAI3 and SOX10. Further validation experiments revealed that ZNF326 is a tumor suppressor gene in TNBC and its knockdown resulted in an expression profile characteristic of an EMT in breast carcinoma cell lines. The effects of ZNF326 knockout to induce an EMT were followed in a panel of fifteen EMT markers [40].

EMT is considered to contribute to metastasis and chemoresistance in patients with hepatocellular carcinoma (HCC), leading to their poor prognosis. To identify genes driving EMT in HCC, immortalized mouse hepatoblasts were subjected to SB mutagenesis. Following transplantation of mutagenized cells to nude mice, mesenchymal liver tumors formed [41]. These tumors were characterized based on their Col1a2, Vim, Fn1, Cdh1, Snai1, Twist1, Zeb1 and Zeb2 expression. However, these tumors also expressed the epithelial markers pan-cytokeratin and Epcam, and no cadherin switch could be identified. These latter findings point toward the induction of an incomplete EMT or a state of epithelial–mesenchymal plasticity in the SB-induced tumor tissues. From the candidate genes identified, three were validated as novel regulators of EMT: knockdown of HUWE1, PTPN12 and KDM6a resulted in the expression of CDH2, FN, VIM and ZEB1 in HCC cell lines. Downregulation of these genes also increased the migratory potential of cells from two HCC cell lines, providing a functional confirmation of the EMT expression profile. Nonetheless, the true extent of EMT induced by these genes is difficult to assess due to the low number of epithelial and mesenchymal markers assessed.

In these studies, the mechanisms of tumorigenesis were studied in mice. The in vivo mutagenesis resulted in the formation of tumors, which were then analyzed to identify the mutations causing these tumors. EMT is also recognized as a central mechanism of tumor cell metastasis. In vivo mutagenesis screens to identify metastasis driver genes are challenging because of the lengthy experiments required until the emergence of harvestable metastatic foci and the complexity given by the constant transposition events ongoing the tumor cells blurring the identification of initial driver mutations. Two in vitro transposon screens were designed to identify metastasis genes.

An in vitro Sleeping Beauty insertional mutagenesis screen was carried out to identify novel genes driving breast cancer metastasis. A non-invasive, non-metastatic breast cancer cell line (SKBR3) was subjected to mutagenesis by introducing the mutagenic load into the cells via repeated transfections. It was hypothesized that the mutagenesis would give rise to a modification in gene expression rendering an invasive phenotype to these cells. Therefore, the mutagenized cell populations were subjected to Boyden chamber-based matrix invasion assays to harvest cells acquiring a de novo invasive phenotype. Following the identification of transposon–host gene fusion transcripts in these cells, two genes were validated as metastasis driver genes. SKBR3 cells overexpressing GIT2 or MUSK acquired an invasive phenotype, as evidenced in a series of in vitro experimental models replicating the different steps of the metastatic cascade: Boyden chamber-based matrix invasion assays, tumor–endothelial cell adhesion assays, transendothelial migration assays, 3D collagen invasion assays and 3D spheroid invasion assays. Additionally, the metastatic potential of GIT2 or MUSK overexpressing cells was also confirmed using two in vivo mouse metastasis models. Moreover, overexpression of both GIT2 and MUSK lead to a significant change in SKBR3 cell morphology characteristic of an EMT. The extent of EMT was followed on a panel of 48 genes. Epithelial markers were downregulated and mesenchymal and myogenic markers were upregulated in GIT2 and MUSK overexpressing SKBR3 cells, corresponding to a completed EMT [42].

In another study designed to identify novel master regulators of colorectal cancer metastasis, a modified, more stringent, in vitro anoikis assay was developed by simultaneously preventing the formation of cell–matrix and cell–cell contacts: cells were seeded on ultralow attachment plates in serum-free medium with the addition of EDTA, a Ca^2+^ chelator, thus disrupting calcium-dependent cell–cell contacts. Additionally, to focus on the mutagenesis events located in the non-coding part of the human genome, the SB transposon used was devoid of the gene-trapping elements used in the studies presented above. The screen was performed in the HCT116 colorectal cancer cell line. It resulted in the isolation of a clone with cells displaying an elongated shape, in contrast to the cuboidal morphology of the initial cells. Cells of this clone were expressing higher levels of mesenchymal markers and decreased E-cadherin expression. As a driver mutation, an insertion located within the 3′UTR of BTB/POZ containing domain protein 7 (BTBD7) was identified, located within the predicted target site of miR-23b, a known anti-metastatic miRNA. BTBD7 is a known EMT regulator, and the newly identified miR-23b::BTBD7 interaction needs to be disrupted in order to allow the onset of EMT and metastasis [43].

## 4. CRISPR-Based Forward Genetic Screens and EMT

Recently, CRISPR-based cancer screens also identified EMT-dependent mechanisms. In a screen to identify genes that promote tumorigenic growth, an immortalized retina pigment epithelial cell line (wild type and TP53^−/−^) was subjected to genome-wide CRISPR knockout screen. Anchorage-independent growth screens and proliferation screens were performed to identify cells with modified phenotypic patterns. The neddylation pathway was highly enriched in the screen. Individual knockout clones of different components of the neddylation pathway (CAND1, UBE2M, UBE2F, CUL5 and CUL3, as well as the CUL3-specific adaptors KCTD10 and KEAP1) were generated. In the TP53^−/−^ background, a change in cell morphology from epithelioid to mesenchymal/spindle shaped was observed, a known characteristic of EMT. Furthermore, loss of CUL3 in TP53-deficient cells resulted in the overexpression of the core EMT transcription factors SNAI2, Twist2, ZEB2, ETS1 and ETS. However, the cells did not fully transition to a mesenchymal state, as the expression of structural markers such as vimentin, fibronectin, E-cadherin or VE-cadherin was unaltered. The CUL3-dependent gene expression changes were, therefore, identified as characteristic for a partial EMT [44].

EMT plays an important role in cancer drug resistance, and two screens revealed relevant aspects for this relationship. In the first screen, mesenchymal cell lines derived from biopsies of non-small-cell lung cancer patients who progressed on EGFR tyrosine kinase inhibitors were subjected to whole-genome CRISPR screening. The goal of screening was to prevent EMT-mediated drug-tolerant cells from surviving and giving rise to resistant clones. FGFR1 was identified to be the top genomic mediator of resistance to third-generation EGFR tyrosine kinase inhibitors. FGFR signaling is necessary for the survival of epithelial, drug-sensitive cells undergoing EMT-like changes during initial exposure to EGFR inhibitors. Dual FGFR + EGFR blockade prevented ERK reactivation that occurred after long-term EGFR inhibitor therapy and consistently suppressed the outgrowth of drug-tolerant clones in multiple EGFR mutant cell line models in vitro, indicating that FGFR signaling is essential for the emergence of mesenchymal-like drug-tolerant clones [45].

Another screen was designed to address novel mechanisms of chemoresistance in pancreatic ductal adenocarcinomas (PDAC). Genome-wide knockout and activation CRISPR screens were used to identify genes that conferred resistance to each of four chemotherapy drugs used in the treatment of PDAC: gemcitabine, irinotecan, 5-fluorouracil and oxaliplatin. Activation of several genes involved in the repression of chromatin via histone deacetylation resulted in multi-drug resistance. Among these genes, HDAC1 overexpression caused an enrichment of certain genes implicated in EMT. Intriguingly, the expression of canonical EMT transcriptional regulators SNAI1, SNAI2 or TWIST1 was not altered, pointing to the involvement of a partial EMT in this phenomenon [46].

One further CRISPR-based screen identified a number of regulatory checkpoints decisive for the acquisition of different EMT or EMP states. In the screen described by Zhang and colleagues, distinct EMT trajectories were identified which may differentially contribute to metastasis. The screen was performed in a phenotypically epithelial subpopulation of HMLER cells by a CRISPR knockout single-guide RNA library targeting over 18,000 genes. A phenotypic selection for mesenchymal cells was carried out by discarding cells that retained a strong epithelial phenotype following two rounds of EpCAM-based magnetic-activated cell sorting and then by CD44-based FACS sorting, achieving a cell population in which 87.9% of cells showed a CD44hi mesenchymal phenotype at day 45. Following the identification of PRC2 and COMPASS epigenetic regulatory complexes, a second CRISPR screen was performed by employing the sgRNA library EPIKOL targeting only genes encoding epigenetic regulators. This latter screen identified sgRNAs targeting the EZH2 and EED genes (encoding two components of the PRC2 complex) as well as the ASH2L gene (encoding a COMPASS component) as being enriched in the emerging mesenchymal populations. The validation experiments suggested that loss of either PRC2 or KMT2D-COMPASS sensitized initially stable epithelial cells to EMT-inducing signals, such as TGF-β. The results also indicated that loss of PRC2 or KMT2D-COMPASS unlocks distinct EMT trajectories and yields two more mesenchymal cell states with strongly differing metastatic abilities. EED-KO quasi-mesenchymal cells, but not parental epithelial cells or the KMT2D-KO highly mesenchymal cells, were able to form macrometastatic colonies in the lung [47].

## 5. Conclusions

While the forward genetic screens described here directly addressed novel EMT genes or EMT mechanisms, a number of further cancer screens did not provide or discuss direct evidence on EMT aspects; however, the individual findings of certain screens are potentially relevant for EMT. For example, loss of LKB1 expression was previously linked to EMT in lung cancer cells [51]. In a recent genome-scale CRISPR screen, LKB1 was identified as a master regulator of chromatin accessibility in lung adenocarcinomas, and the loss of LKB1 activated SOX17 in metastasis and in a metastatic-like subpopulation of cancer cells within primary tumors [52]. Similarly, the known EMT regulator NFIB [53] was identified in an inducible piggyBac transposon mutagenesis screen to promote breast cancer metastasis via the NFIB-ERO1A axis [54]. Analyzing further forward genetic screens could reveal additional findings and correlations regarding EMT. Moreover, interesting tools are available, which could contribute to the reevaluation of earlier, potentially EMT-relevant, datasets. The Sleeping Beauty Cancer Driver Database contains *Sleeping Beauty* transposon insertion data derived from 2354 tumors representing 19 distinct mouse models of human cancer [55]. Similar databases were compiled to process the massive amounts of data generated with CRISPR screens. For example, GenomeCRISPR contains data derived from 84 different experiments performed in 48 different human cell lines, allowing the investigation of phenotypic correlations [56]. BioGRID, the database of protein–protein interactions, recently opened a new dataset of CRISPR phenotype screens in human and mouse cell lines [57]. The DepMap database was generated to define a cancer dependency map, and is based on genome-scale CRISPR–Cas9 essentiality screens across 342 cancer cell lines [58]. Further tools (such as the iCSDB, an integrated database of CRISPR screens [59]) were generated which combine the data contained in other databases and add subsequent layers of phenotype endpoints, giving the investigator multiple choices to gain more exact and tailored results.

Before embarking on a forward genetic screen, there are some aspects to be pondered regarding the advantages and disadvantages of the screening systems. For example, the CRISPR system may present the advantage of inflicting a wider range of mutation types, targeting both alleles. Nonetheless, in vivo CRISPR screens are more challenging. Advantages of transposon screens may include in vivo tissue-specific mutagenesis and the possibility to identify regulatory regions of the genomes relevant for a specific phenotype, yet within a reduced mutation spectrum [20,23].

As indicated above while discussing some of the findings, the extent of characterization of EMT features is essential when interpreting findings of forward genetic screens and the subsequent validation experiments. Recommendations on the criteria to define EMT were formulated recently. Accordingly, it is insufficient to assess EMT based on one or a small number of markers. A combination of markers and changes in cellular properties should rather be used to define EMT [60]. This approach was only applied in a subset of the studies reviewed here.

Forward genetic screens represent powerful tools to further extend our understanding of the complexity of cancer development, progression or response to therapy, and EMT is at the forefront of these mechanisms. In this review, we covered some of the contributions of such cancer screens made to the field of EMT. We propose forward genetic screens, and, in particular, transposon insertional mutagenesis and CRISPR-based screens, as powerful resources to identify additional mechanisms of EMT in cancer, which may also bear further relevance for EMT in other pathological conditions. The expanding knowledge on the role and mechanisms of EMT during human disease may pave the way for new treatment strategies to reduce the mortality and morbidity of patients.

## Figures and Tables

**Table 1:** Genome-wide transposon-based forward genetic screens identifying the roles of EMT and EMT genes.

Genes Identified	Transposon (Sleeping Beauty)	Mutagenesis	Phenotype Selection	Phenotype Readout	Tumor Entity	Species	Genetic Background	Reference
NOTCH1	T2Onc3	In vivo	In vivo	Tumorigenesis	TNBC	mouse	BRCA1 mutant	[38]
TRPS1	T2Onc2, T2Onc3	In vivo	In vivo	Tumorigenesis	TNBC	mouse	PTEN mutant	[39]
ZNF326	T2Onc2, T2Onc3	In vivo	In vivo	Tumorigenesis	TNBC	mouse	PTEN mutant	[40]
MET GAB1 HUWE1 KDM6A PTPN12	T2Onc2	In vitro	In vivo	Tumorigenesis	HCC	mouse	-	[41]
GIT2 MUSK	T2Onc3	In vitro	In vitro	Metastasis (matrix invasion assay)	Breast cancer	human (SKBR3 cell line)	-	[42]
miR-23b::BTBD7	pT2-CMV-EGFP	In vitro	In vitro	Metastasis (forced single cell suspension)	Colorectal cancer	human (HCT116 cell line)	-	[43]

**Table 2:** Genome-wide CRISPR-based forward genetic screens identifying the roles of EMT and EMT genes.

Genes Identified	CRISPR Library Type	Mutagenesis	Phenotype Selection	Phenotype Readout	Tumor Entity	Species	Genetic Background	Reference
CUL3	GeCKOv2 (CRISPR knockout)	In vitro	In vitro	Tumorigenesis: anchorage-independent growth screens by soft agar assay, proliferation screens	-	human	TP53-deficient	[44]
FGFR1	whole-genome CRISPR knockout screening	In vitro	In vitro	Drug resistance	NSCLC	human	EGFR mutant	[45]
HDAC1	GeCKOv2 (CRISPR knockout), SAMv1 (Human CRISPR Activation Library)	In vitro	In vitro	Drug resistance	Pancreatic ductal adenocarcinoma	human	-	[46]
PRC2 KMT2D-COMPASS	CRISPR Knockout Library	In vitro	In vitro	Mesenchymal morphology	Breast cancer	human	-	[47]
